# Molecular function and potential evolution of the biofilm-modulating blue light-signalling pathway of *Escherichia coli*

**DOI:** 10.1111/j.1365-2958.2012.08147.x

**Published:** 2012-07-12

**Authors:** Natalia Tschowri, Sandra Lindenberg, Regine Hengge

**Affiliations:** Institut für Biologie – Mikrobiologie, Freie Universität Berlin14195 Berlin, Germany

## Abstract

*Escherichia coli* senses blue light via the BLUF-EAL protein BluF (YcgF). The degenerate EAL domain of BluF does not have cyclic-di-GMP phosphodiesterase activity, but BluF directly antagonizes the MerR-like repressor BluR (YcgE), which leads to expression of the *ycgZ-ymgABC* operon and activation of the Rcs system (Tschowri *et al*., 2009; *Genes Dev* 23: 522–534). While *bluR*, *bluF* and *ycgZ* have individual transcriptional start sites, comparative genome analysis indicates that the *bluR-bluF-ycgZ-ymgAB* region represents a functional unit in various enteric bacteria that is characterized by *bluF* alleles encoding degenerate EAL domains. Re-introducing conserved amino acids involved in phosphodiesterase activity of EAL domains did not restore enzymatic activity or c-di-GMP binding of BluF, but weakened its ability to antagonize BluR and improved a residual interaction with the BluR paralogue MlrA, which controls expression of the biofilm regulator CsgD and curli fibres. We identified the BluR binding site in the *ycgZ* promoter and observed that BluR also has residual affinity for the MlrA-dependent *csgD* promoter. Altogether, we propose that BluF evolved from a blue light-regulated PDE into a specific antagonist of a duplicate of MlrA that became BluR, which controls not only curli but various biofilm functions via the Ymg/Rcs pathway.

## Introduction

*Escherichia coli* is a Gram-negative enterobacterium, which can switch between host-associated and environmental lifestyles. It exists in the mammalian intestine as well as under outside conditions, e.g. in aquatic milieus or in soil. In its natural outside environment *E. coli* is able to sense and to respond to blue light via the photoreceptor protein YcgF, which carries an N-terminal BLUF [*b*lue *l*ight *u*sing *F*AD (flavin adenine dinucleotide)] domain (Gomelsky and Klug, 2002; Rajagopal *et al*., 2004; Nakasone *et al*., 2007; 2010). The BLUF domain of YcgF is associated with a C-terminal EAL domain. In general, EAL domain-containing proteins act as phosphodiesterases (PDE) that degrade the biofilm-promoting second messenger c-di-GMP (Hengge, 2009; Schirmer and Jenal, 2009). However, all four amino acids known to play a key role in c-di-GMP binding of EAL domains as well as an essential catalytic glutamic acid and other amino acids that contribute to PDE activity (Rao *et al*., 2008) are not conserved in YcgF of *E. coli* (Fig. S1). Consistently, in our previous study (Tschowri *et al*., 2009) we demonstrated that YcgF does not bind or degrade c-di-GMP irrespective of blue light irradiation. Instead, YcgF directly binds to the MerR-like repressor YcgE and releases it from its operator DNA in a light-dependent manner. Inactivation of YcgE results in elevated expression of the *ycgZ-ymgABC* operon, which is under direct control of the YcgE repressor protein and located right next to *ycgE-ycgF* on the *E. coli* chromosome. The YcgF/YcgE controlled YmgB protein and, to some extent also YmgA, can modulate biofilm functions by activating the Rcs phosphorelay system, which results in increased colanic acid production and a downregulation of curli fibre synthesis (Tschowri *et al*., 2009).

YcgE represents a closely related paralogue of MlrA, a MerR-like regulator that directly activates the transcription of the important biofilm regulator CsgD (Brown *et al*., 2001; 2003; Ogasawara *et al*., 2010). In *E. coli* and other enteric bacteria CsgD was shown to positively regulate the synthesis of *csgBAC*-encoded curli fibres (Römling *et al*., 1998; 2000; Brombacher *et al*., 2003). To activate *csgD*, MlrA cooperates with the phosphodiesterase YciR and the diguanylate cyclase YdaM (Weber *et al*., 2006), with these three proteins showing multiple direct interactions (S. Lindenberg and R. Hengge, unpubl. data).

The strong sequence conservation of both domains in YcgE and MlrA (Fig. S2) and the observation that both proteins interact with EAL-domain proteins, suggest that the two proteins have a direct common ancestor. In addition, YcgF, which is present in a variety of bacterial species, occurs in different ‘evolutionary intermediates’ between an active PDE and an anti-repressor protein (Fig. S1). The YcgF protein from *E. coli* is the most degenerate variant with respect to residues essential for PDE activity (Tschowri *et al*., 2009), whereas BlrP1, which is one of two YcgF homologues in *Klebsiella pneumoniae*, was shown to possess blue light-regulated phosphodiesterase activity (Barends *et al*., 2009). Altogether, it seems obvious that recent evolution has occurred in the *ycgE-ycgF-ycgZ-ymgABC* genomic region and with this study we further characterize the expression and molecular functions of the components of the YcgF–YcgE–Ymg pathway, also with the intention to gain insight into its potential evolution.

Here we show that the *ycgE-ycgF-ycgZ-ymgAB* region represents a functional unit conserved in various enteric bacteria. Comparative genomic analyses revealed that YcgF homologues encoded within this genetic unit usually show a certain degree of degeneration. Moreover, some species, e.g. *Klebsiella pneumonia*, encode for an additional enzymatically active YcgF variant, which suggests that YcgF originally evolved from an active PDE following gene duplication. Yet, re-introducing all amino acids typically required for PDE activity did not restore enzymatic activity or c-di-GMP binding of *E. coli* YcgF, but compromised its potential to antagonize YcgE, thus demonstrating that YcgF is not just a defective PDE but specifically adapted to interact with YcgE. On the other hand, restoration of consensus amino acids in the degenerate EAL domain of YcgF improved a residual affinity for the YcgE paralogue MlrA. Moreover, YcgE was shown to have residual binding ability to the MlrA-controlled *csgD* promoter.

Finally, in response to requests by other researchers for more meaningful gene designations, we now also propose to rename these genes, i.e. ‘*bluF*’ for *ycgF* and ‘*bluR*’ for *ycgE* and will use these designations in the following.

## Results

### Genetic organization of the *bluR-bluF-ycgZ-ymgABC* region on the *E. coli* chromosome

**Fig. 1:**
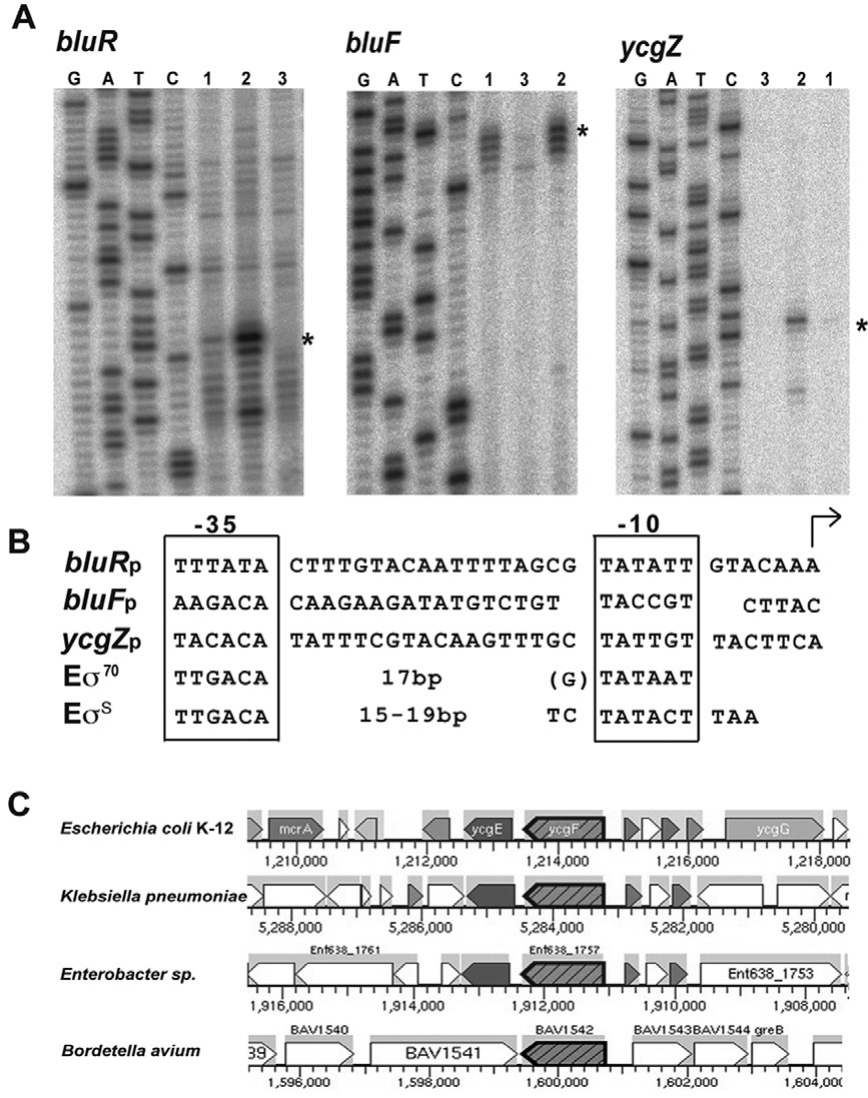
Genetic organization and transcriptional start sites of *bluR*, *bluF* and the *ycgZ-ymgABC* operon. A. Determination of the transcriptional start sites of *bluR*, *bluF* and the *ycgZ-ymgABC* operon by primer extension. MC4100 wild-type cells (lanes 1) and derivatives containing plasmids carrying the promoter regions of *bluR*, *bluF* and *ycgZ* (lanes 2) or the corresponding knockout mutations (lanes 3) were subject to RNA isolation and primer extension as described in the *Experimental procedures*. The longest reverse transcripts that were present in higher amounts with the plasmid-containing strains and absent with the mutants represents the transcriptional start points and are highlighted with an asterisk (*). B. Sequences of the *bluR*, *bluF* and *ycgZ* promoter regions. The putative −35 and −10 regions as well as the Eσ^70^ and Eσ^70^ consensus sequences are boxed, transcriptional start sites are indicated with the arrow. C. Genetic organization of the *bluR-bluF*-*ycgZ-ymgABC* region of *E. coli* in comparison with corresponding regions of *Klebsiella pneumoniae*, *Enterobacter* sp*.* and *Bordetella avium* obtained with the Multi-Genome alignment tool provided by EcoCyc.

Via direct protein–protein interaction, the BluF (YcgF) protein of *E. coli* interferes with the binding of the MerR-like protein BluR (YcgE) to the *ycgZ* promoter in a blue light-dependent manner (Tschowri *et al*., 2009). *ycgZ* is the first gene of the *ycgZ-ymgA-ymgB-ymgC* operon, which encodes four small proteins and is located adjacent to the *bluR-bluF* region separated by a divergently transcribing control region (Fig. 1C). Northern blot analysis indicated that *ycgZ-ymgA-ymgB-ymgC* are expressed in a single polycistronic mRNA. Moreover, based on reporter gene fusion experiments (Tschowri *et al*., 2009) we concluded that *bluR* and *bluF* do not constitute an operon. In this study we complemented these data by determining the transcriptional start sites of *bluR*, *bluF* and *ycgZ*.

The 5′-mRNA ends for *bluR*, *bluF* and *ycgZ* identified by primer extension experiments (Fig. 1A and B) are located 54, 30 and 36 nucleotides, respectively, upstream of the corresponding translational start sites. These results demonstrate that *bluR* and *bluF* are transcribed independently. Consistent with the consensus sequences of σ^70^- and σ^S^-dependent promoters (Fig. 1B) (Typas *et al*., 2007), *bluR* and *bluF* are known to be transcribed by σ^70^-containing RNA polymerase (RNAP), whereas the expression of the *ycgZ-ymgABC* operon is under σ^S^ control (Tschowri *et al*., 2009). With 18 bp, the spacing between the −35 and −10 elements in the promoter region of the BluR-regulated gene *ycgZ* is consistent with recognition by σ^S^-containing RNAP as well as with control by a MerR-like regulator, which usually bind overlapping with promoter regions that exhibit spacer lengths greater than 17 bp (Brown *et al*., 2003).

Knowing that *bluR-bluF-ycgZ-ymgABC* act in a common regulatory pathway we wondered, whether this functional genetic unit is conserved in other enteric bacteria. Using the Multi-Genome alignment tool provided by EcoCyc (Keseler *et al*., 2011) and blast (Altschul *et al*., 1997) we found that even closely related species differ in the number of BluF homologues they contain. Whereas none of the currently sequenced *Yersinia* and *Salmonella* species has such a photoreceptor protein, *E. coli* K-12 and *Citrobacter* species possess one BluF protein each and *Klebsiella* as well as *Enterobacter* species even have two versions of BluF. Furthermore, BluF proteins present in these enteric bacteria show different degrees of degeneration of the EAL domain with regard to key amino acids essential for c-di-GMP-dependent phosphodiesterase activity (Rao *et al*., 2008), with BluF from *E. coli* representing the most degenerate variant (Fig. S1).

The KPK_2789 protein (also called BlrP1) from *K. pneumoniae* displays all residues essential for PDE activity and was in fact shown to act as a blue light-regulated PDE (Barends *et al*., 2009). In addition, *K. pneumoniae* possesses a second BluF homologue, KPK_3794, which is encoded next to the gene for KPK_3793 (Fig. 1C). The latter protein is annotated as ‘MlrA’, but in fact shows 65% identity to BluR and 48% identity only to MlrA from *E. coli*, whereas another MerR-like protein (KPK_4910) in the *K. pneumoniae* chromosome shows 37% identity to BluR and 44% identity to MlrA. Interestingly, the BluF homologue KPK_3794, which forms a coding unit with the more BluR-like KPK_3793, carries a partially degenerate EAL domain that is missing an aspartic acid involved in c-di-GMP binding as well as a glutamic acid involved in binding of the cofactor Mg^2+^. Moreover, this genetic unit is associated with a small operon related to the *ycgZ-ymgAB* region from *E. coli* (without *ymgC*; Figs S1 and 1C).

A similar situation is found in *Enterobacter* sp. 638, which not only has two BluF homologues, but also two BluR-related MerR-like proteins. Among the two BLUF-EAL proteins, Ent638_2032 shows a canonical EAL domain protein that most likely acts as a PDE, whereas Ent638_1757 is degenerate to some extent and forms a coding unit with the BluR homologue Ent638_1758 and *ycgZ-ymgAB*-like small genes (Fig. 1C). Finally, *Bordetella avium* and *Alteromonas macleodii*, which do not have any BluR homologues but carry genes for BluF-like proteins with consensus EAL domains and therefore most likely PDE activity, stand for the other extreme, i.e. for species that seem to have a blue light-regulated PDE, but do not feature BluR and its target operon *ycgZ-ymgAB*.

Taken together, these observations show that different evolutionary intermediates of BluF exist in bacteria and that whenever a *bluF* homologue encoding for a protein with a *degenerate* EAL domain exists in a bacterial genome, it is usually located next to the gene for a MerR-like BluR-related protein and a *ycgZ*-*ymgAB*-like genetic unit (Fig. 1C).

### Restoration of consensus amino acids in the degenerate EAL domain of BluF does not reconstitute PDE activity but reduces its ability to antagonize BluR

With BluF existing in different intermediate variants between an active PDE (as BlrP1 in *K. pneumoniae*) and a degenerate EAL domain protein now acting as an anti-repressor (as in *E. coli*), we wondered whether it is possible to perform ‘reverse evolution’ and to mutate the *E. coli* BluF protein ‘back’ to an active PDE by introducing the key amino acids required for enzymatic activity (see Fig. S1). We therefore generated a series of BluF mutant variants with increasing similarity to a consensus EAL domain (M2–M8, see *Experimental procedures*). The purified proteins were tested for phosphodiesterase activity and c-di-GMP binding ability using radiolabelled c-di-GMP *in vitro*. In contrast to the active phosphodiesterase YhjH (Pesavento *et al*., 2008) or the diguanylate cyclase PleD* (Chan *et al*., 2004), none of the BluF variants was able to cleave or bind c-di-GMP (Fig. S3A and B).

These mutant BluF versions were also tested for their ability to degrade c-di-GMP *in vivo*. For this purpose a *yhjH::kan* mutant derivative of W3110, which is compromised in motility (Fig. S3C) due to elevated cellular c-di-GMP levels (Pesavento *et al*., 2008), was transformed with pQE30Xa derivatives encoding the different BluF variants. Reduced motility can be suppressed by expression of YhjH (even from the low-copy-number plasmid pCAB18 as in Fig. S3C) or of another active PDE, e.g. YciR (C. Pesavento and R. Hengge, unpubl. results). Accordingly, we expected any plasmid-encoded enzymatically active BluF mutant variant to suppress the non-motile phenotype of a *yhjH* mutant. However, none of the mutant BluF proteins expressed from pQE30Xa was able to restore motility of the *yhjH* mutant (Fig. S3C). All these results demonstrate that introducing key amino acids that contribute to binding and cleavage of c-di-GMP as well as to binding of Mg^2+^ is not enough to restore PDE activity in BluF.

**Fig. 2:**
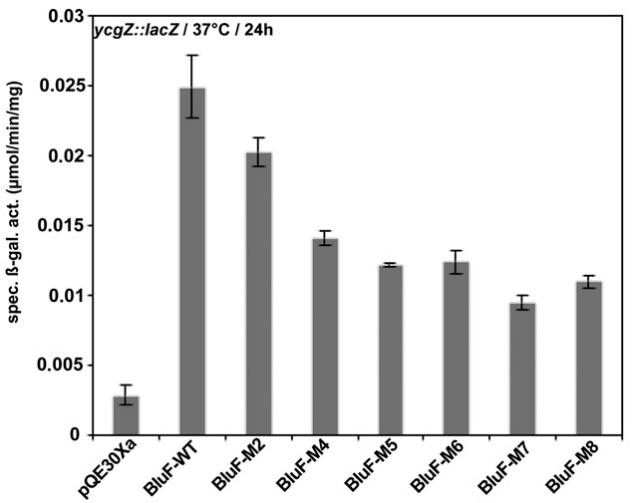
Restoration of consensus amino acids in the EAL domain reduces the ability of BluF to derepress *ycgZ::lacZ* expression. Expression of a single-copy chromosomal *ycgZ::lacZ* fusion was determined in W3110 derivatives carrying pQE30Xa-encoded wild-type BluF as well a mutant variants (for nomenclature of mutations, see *Experimental procedures*). Cells were grown at 37°C in LB/ampicillin for 24 h and specific β-galactosidase activities were determined.

But do these amino acid exchanges in BluF alter its ability to antagonize the repressor protein BluR? To test this, the same pQE30Xa-encoded BluF variants were expressed in a W3110 derivative carrying a *ycgZ::lacZ* reporter gene fusion, which represents a target gene under BluF/BluR control, and were tested for their potential to derepress *ycgZ::lacZ*. As shown in Fig. 2, only the least mutated BluF-M2 (BluF^I193L+Q195R^) variant, in which the degenerate motif EAIVQ was replaced by the consensus signature EALVR, was still able to derepress the *ycgZ::lacZ* expression almost to the same extent as wild-type BluF expressed from the same vector. The BluF variants with higher numbers of amino acid exchanges (M4–M8) showed reduced ability to derepress *ycgZ::lacZ* and therefore to antagonize BluR, although they were expressed at the same levels as the wild-type protein (data not shown).

In conclusion, re-introducing amino acids typically conserved in enzymatically active EAL domain does not restore PDE activity of BluF, but rather compromises its ability to counteract BluR. Thus, BluF is not just a defective PDE but has been evolutionarily adapted to bind and antagonize BluR.

### BluF has residual affinity for the BluR paralogue MlrA which is improved upon restoring of consensus amino acids in the degenerate EAL domain

**Fig. 3:**
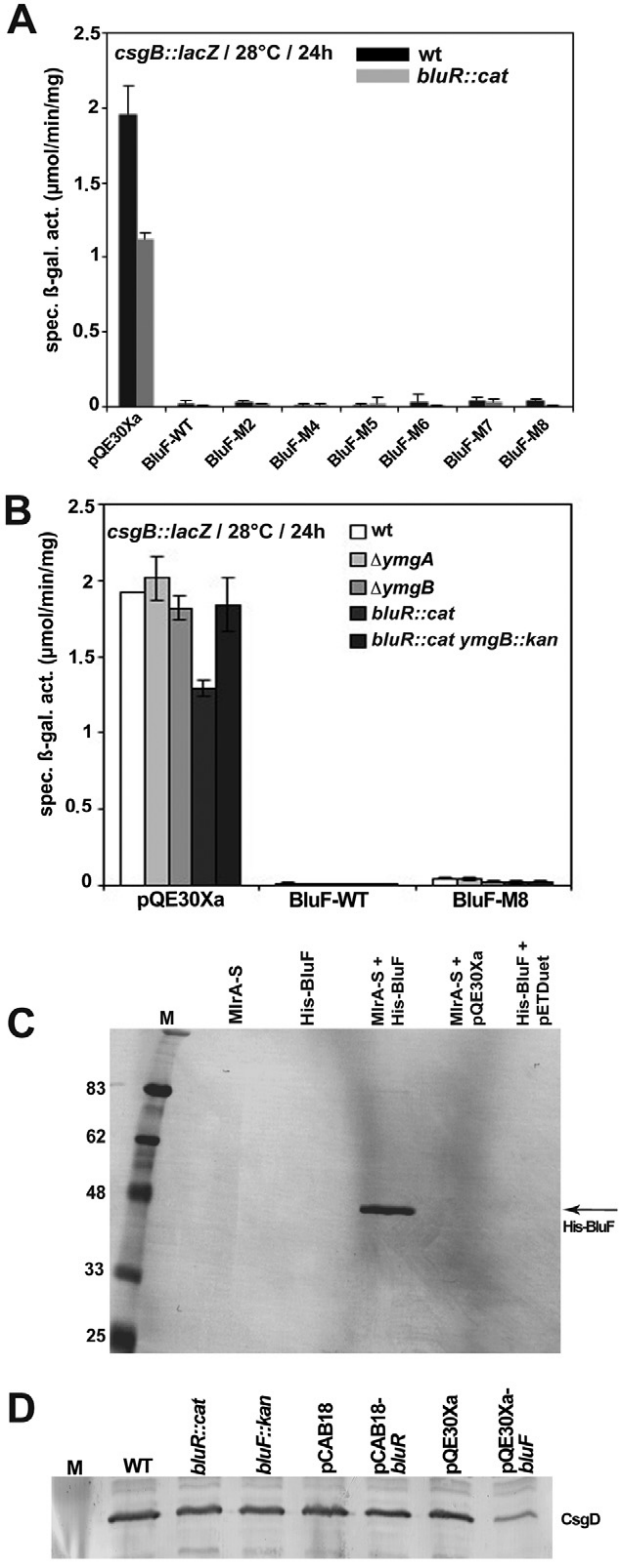
BluF represses CsgD and curli expression when overexpressed *in vivo* and interacts with MlrA *in vitro*. A. Overexpressed wild-type BluF and its mutated variants downregulate *csgB::lacZ* expression independently of BluR. Wild-type W3110 and its *bluR::cat* derivative were transformed with pQE30Xa derivatives expressing wild-type BluF as well as the indicated mutant variants. β-Galactosidase activities were determined after 24 h growth in LB/ampicillin at 28°C. B. Overexpressed wild-type BluF and BluF-M8 downregulate *csgB::lacZ* independently of YmgA or YmgB. The assay was performed as described under (A), except that the indicated mutant backgrounds were used. C. BluF forms a complex with MlrA *in vitro*. Using an extract of total soluble cellular proteins, S-tagged MlrA was bound to S-protein agarose by affinity chromatography and mixed with a second extract containing His-tagged BluF. Retention and co-elution of BluF was detected by immunoblotting using an anti-His antibody. D. Cellular levels of CsgD in W3110 (WT) and its *bluR::cat* and *bluF::kan* derivatives as well as in W3110 overexpressing BluR or BluF or carrying the corresponding empty vectors (pCAB18 and pQE30Xa). Cells were grown in LB medium containing ampicillin (for plasmid-containing strains). No inducer (IPTG) was added. At an OD_578_ of 3 cells were harvested for immunoblot analysis.

Knocking out *bluF* in *E. coli* does not influence the c-di-GMP-responsive expression of curli fibres as monitored by a *csgB::lacZ* reporter gene fusion (Sommerfeldt *et al*., 2009). On the other hand, a *bluR* mutant shows a reduction in curli expression due to enhanced expression of YmgB which activates the RcsDBC phosphorelay system (Tschowri *et al*., 2009). Increased activity of the Rcs system results in elevated expression of the small RNA RprA, which interferes with the translation and reduces the cellular mRNA level of *csgD*, encoding the major activator of the *csgBCA* operon (Jørgensen *et al*., 2012; Mika *et al*., 2012). Surprisingly, we observed that moderate overproduction of BluF and even of those BluF-M2–M8 variants, which were unable to fully antagonize BluR, strongly downregulated curli expression (Fig. 3A). Moreover, this effect was much stronger than that of knocking out BluR and occurred also in strain in which *bluR* (Fig. 3A) or the target genes *ymgA* or *ymgB* were absent due to mutations (Fig. 3B), suggesting that increased amounts of BluF can downregulate curli expression in a way that bypasses the BluR-regulated pathway.

Since the expression of the curli regulator CsgD is under the control of the BluR paralogue MlrA (Brown *et al*., 2001; Ogasawara *et al*., 2010), we hypothesized that BluF, when present at elevated levels, may be able to bind MlrA and thereby also act as an anti-activator to MlrA and *csgD* transcription. To test this hypothesis we performed protein–protein interaction analysis *in vitro* using affinity chromatography (‘pull-down’) with extracts from cells expressing either the S-tagged MlrA or the His6-tagged BluF. Due to the presence of other cellular proteins only specific interactions should be detected. The S-tag served for affinity chromatography to bind MlrA to S-protein agarose. As detected by immunoblot analysis with a His6-tag antibody, His6-tagged BluF indeed co-eluted after incubation of the S-tagged MlrA-containing extract with lysate from His6-tag-BluF-expressing cells (Fig. 3C). Thus, BluF interacts with MlrA *in vitro*, suggesting that BluF, when overexpressed *in vivo*, can interfere with *csgD* expression by antagonizing MlrA. Consistently, overexpression of BluF resulted in decreased levels of CsgD (Fig. 3D).

MlrA and BluR have a characteristic MerR-like DNA-binding N-terminal domain (NTD) and also share a specific C-terminal domain (CTD; Fig. S2). In classical MerR-like proteins the CTD serves as the ligand-binding domain (Brown *et al*., 2003). Affinity chromatography with purified proteins has previously shown that *in vitro* BluF interacts with the DNA binding BluR^NTD^ (Tschowri *et al*., 2009). In order to confirm the interaction between BluF and MlrA also *in vivo* and to assign this interaction to a specific domain of MlrA, we used the BacterioMatch II Two-Hybrid System. In this system, the two potentially interacting proteins or domains are expressed as fusion proteins to the NTD of the cI repressor of phage lambda (from the pBT vector) and to the NTD of the alpha subunit of *E. coli* RNAP (from the pTRG vector). After co-transformation, interaction of the two fusion proteins enables the histidine-auxotrophic *E. coli* reporter strain to grow on selective plates due to increased expression of the yeast His3 gene (Dove and Hochschild, 2004). For comparison, we also included the N-terminal as well as the C-terminal domains of BluR in this *in vivo* interaction analysis. Elevated expression of BluR is toxic for *E. coli* (Tschowri *et al*., 2009) and therefore, full-size BluR could not be tested in these interaction assays. Wild-type BluF as well as the BluF-M8 variant (which carries I193L, Q195R, M362E, A365E, T247N, H177Q, H306D, S328D and therefore best resembles a canonical EAL domain protein) served as bait proteins in this experimental set-up.

**Fig. 4:**
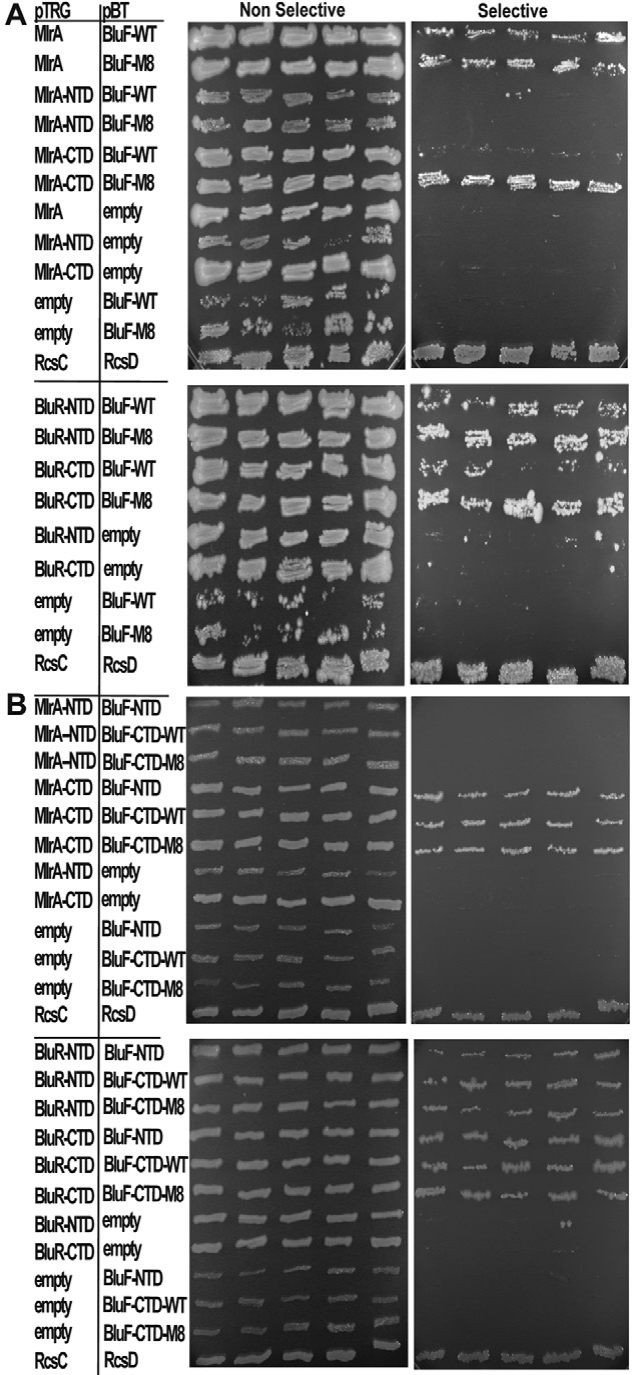
Two-hybrid analysis reveals that restoration of consensus amino acids in the EAL domain of BluF improves *in vivo* binding to MlrA^CTD^ and that BluF interacts with N- and C-terminal domains of BluR. Using the Bacterio-Match two-hybrid system, reporter cells were co-transformed with derivatives of the pBT and pTRG plasmids and appropriate vector only controls. (A) Wild-type (WT) BluF and BluF-M8 as well as (B) the NTD and EAL domains alone of BluF and BluF-M8 were expressed as hybrid proteins fused to cI^NTD^ on pBT. MlrA and BluR as well as their NTD or CTD alone were synthesized from pTRG as fusions to RNAP alpha-NTD. The previously known interaction between the cytosolic part of the RcsC kinase and the soluble fraction of the phosphotransmitter protein RcsD served as positive control (Takeda *et al*., 2001). Interaction is detected by growth in the presence of the His3 inhibitor 3-AT (‘selective’) at 37°C for 24 h following incubation at 28°C for 48 h. Each row on the plates shows patches of five independent co-transformants of the protein or domain combinations indicated. As a second readout of the assay, numbers of co-transformants able to grow on selective screening medium normalized to numbers on non-selective medium were determined (shown in Fig. S4).

The two-hybrid system confirmed the interaction between MlrA and wild-type BluF (Fig. 4A) first observed *in vitro* (Fig. 3C). Moreover, this assay showed that BluF interacted with the C-terminal domain of MlrA, which at first glance seemed surprising given the previous result that BluF interacts with the N-terminal domain of the MlrA paralogue BluR (Tschowri *et al*., 2009). However, the two-hybrid analysis not only confirmed this BluF–BluR^NTD^ interaction but also revealed a weaker interaction of BluF with the BluR^CTD^ (Fig. 4A). Two-hybrid assays with the isolated N-terminal and EAL domains of BluF showed that both BluF domains contribute to the interactions with MlrA and BluR, and confirmed that only MlrA^CTD^ is contacted whereas in BluR, both domains are involved (Fig. 4B).

Finally, it was observed that the BluF-M8 variant (the full size protein) showed stronger interaction with MlrA than wild-type BluF. This became particularly apparent with the MlrA^CTD^ alone, which hardly interacted with wild-type BluF, but showed clear interaction with BluF-M8 (Fig. 4A). In that respect, introducing the mutations present in BluF-M8 resulted in a gain of function. In contrast, the isolated EAL domains of BluF and BluF-M8 both interacted equally well with all interaction partner domains (Fig. 4B), suggesting that in wild-type BluF the sites of interaction with MlrA are present but conformationally occluded in a way that is relieved in the mutated BluF-M8 variant.

### BluR does not only bind to the *ycgZ* promoter region, but also shows residual binding to the *csgD* promoter region

BluR and MlrA are not only MerR-like regulators (which are defined by similar NTDs), but rather are direct paralogues that also share the CTD, which results in the same length of 243 amino acids as well as 49% overall amino acid identity (Fig. S2). In comparison, BluR shows only low overall identity to other MerR-like proteins of *E. coli* (12% to SoxR, 17% to CueR and 14% to ZntR) (Fig. S2). Moreover, BluR and MlrA have similar N-terminal DNA-binding domains with only four residues differing in the helix–turn–helix motif. The C-terminal putative ligand-binding domain, which is known to define the specificity of MerR-like regulators (Brown *et al*., 2003), is still 37% identical between the two proteins. Thus, BluR and MlrA have most likely evolved from a common ancestral gene, possibly involving a gene duplication event.

**Fig. 5:**
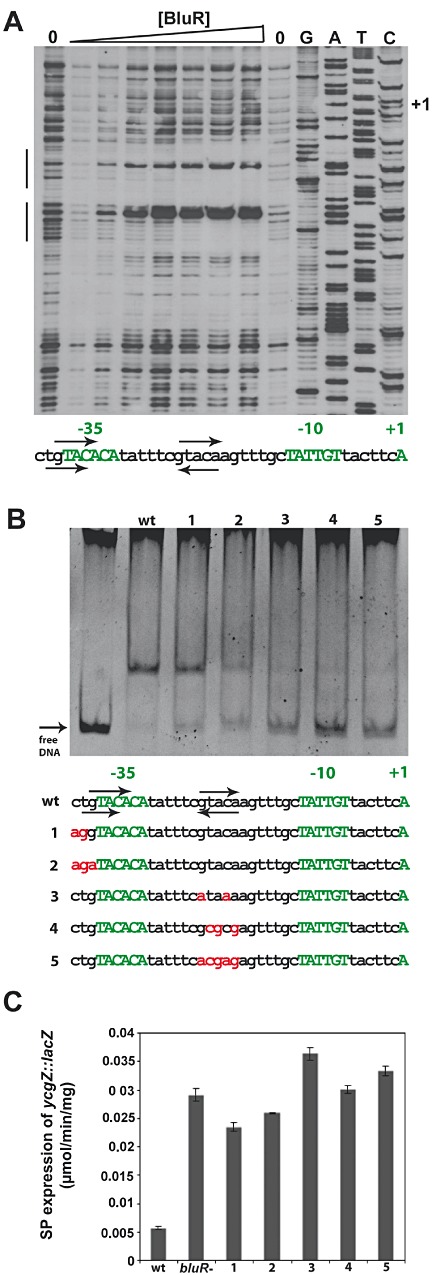
The BluR binding site in the *ycgZ* promoter. A. BluR binding sites in the *ycgZ* promoter were determined by DNase I footprint analysis using purified BluR and a Digoxigenin-labelled DNA fragment carrying the *ycgZ* promoter region. Vertical lines show the protected region, ‘+1’ indicates the transcriptional start site. BluR binding sites were mapped to the *ycgZ* promoter sequence and pointed out with arrows (arrows in the same direction indicate a direct sequence repeat, arrows pointing towards each other an inverted repeat). B. The indicated mutations (red) were introduced into the BluR binding region of the *ycgZ* promoter and BluR binding was tested *in vitro* by EMSA (both in the sequence and in the shift assay mutations are indicated by numbers 1–5). C. Stationary-phase expression of single-copy *ycgZ::lacZ* fusions containing wild-type (wt) or mutated BluR binding sites (1–5) in the W3110 background. For comparison, also a *bluR::cat* derivative of W3110 carrying the wild-type *ycgZ::lacZ* fusion is shown. Cells were grown in LB at 37°C and β-galactosidase activities were determined in triplicate after entry into stationary phase of growth.

The MlrA binding site in the *csgD* promoter region was recently identified (AAAGTT**GTACA**(12N)T**GCACA**ATTTT) (Ogasawara *et al*., 2010). Here we determined the BluR binding site on the *ycgZ* promoter, so the recognition sites of these closely related proteins can be compared. DNase I footprint analysis was performed using purified tag-free BluR and a *ycgZ* promoter-containing DNA fragment. A protected site was identified (Fig. 5A) in which a repetitive motif (GTACA. … GTACA) directly overlaps with a palindromic motif (TGTAC. … GTACA) (see arrows in Fig. 5B). This site is located within the *ycgZ* promoter, i.e. BluR binds to two half-sites overlapping with the −35 region and the spacer region of the promoter. Interestingly, the GTACA motif recognized by BluR is also present in the MlrA binding site in the *csgD* promoter region (see above, highlighted in bold). Two DNase I-hypersensitive sites within the BluR-protected region (Fig. 5A) indicate that BluR binding also results in DNA bending.

To further confirm a role for this BluR binding site in the *ycgZ* promoter, different point mutations were introduced into the two half-sites of the binding site (labelled in red in Fig. 5B). These mutations resulted in reduced or even a loss of DNA binding by BluR *in vitro* as shown in an electrophoretic mobility shift assay (Fig. 5B). Moreover, when introduced *in vivo* into the promoter region of a single-copy *ycgZ*::*lacZ* fusion, these mutations derepressed *ycgZ* expression to the same extent as did a *bluR::cat* knockout mutation, indicating that they abolished the ability of BluR to repress *ycgZ* expression (Fig. 5C). Altogether, these data confirmed the BluR binding site in the *ycgZ* promoter region identified by DNase I footprinting.

**Fig. 6:**
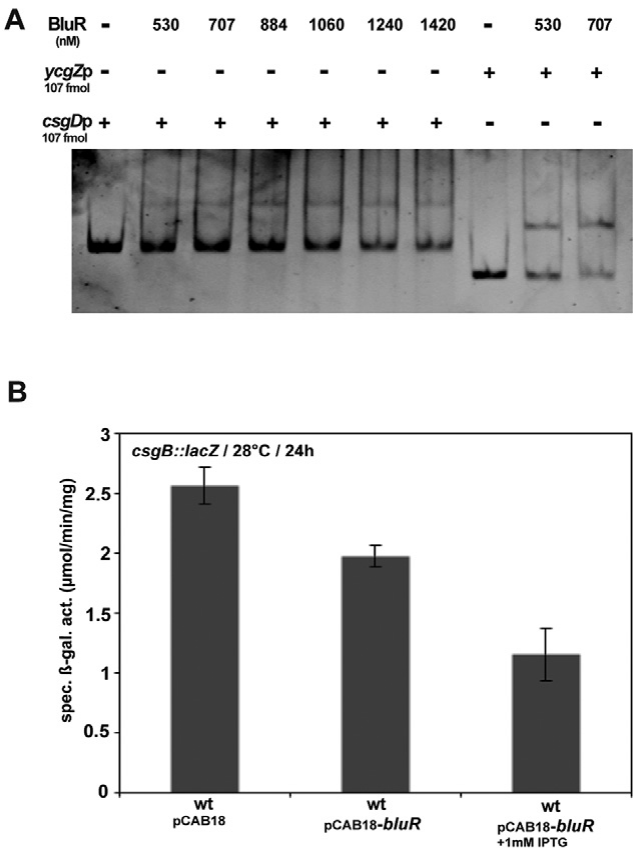
Binding of BluR to the *ycgZ* and *csgD* promoter regions. A. Binding of BluR to the *ycgZ* and *csgD* promoter regions was compared by EMSA. DNA fragments containing either the *ycgZ* promoter (158 bp) or the *csgD* promoter region (198 bp) were incubated with purified BluR at concentrations as indicated and run on a 5% polyacrylamide gel prior to ethidium bromide staining. B. Expression of a single-copy chromosomal *csgB::lacZ* fusion in W3110 expressing BluR from the low-copy-number plasmid pCAB18 or containing the empty vector only. Cells were grown at 28°C in LB/ampicillin in the presence or absence of 1 mM IPTG as indicated for 24 h and specific β-galactosidase activities were determined.

Since BluR has a similar DNA-binding helix–turn–helix motif (Fig. S2) as MlrA and binds to a GTACA sequence motif, which is also present in the MlrA binding site in the *csgD* promoter region, we wondered whether BluR is able to bind to the *csgD* promoter region, too. In an electrophoretic mobility shift assay, BluR could indeed bind to a *csgD* promoter fragment, albeit with lower affinity than to the *ycgZ* promoter fragment (Fig. 6A). Interestingly, BluR could also downregulate *csgD* and curli expression when expressed from a plasmid, especially when inducer was added (Fig. 6B). In summary, BluR may have evolved from a duplicate of MlrA, as is suggested not only by its structural and functional similarity to MlrA, but is also evident from its residual ability to directly bind to the MlrA-dependent *csgD* promoter, which – when overproduced – allows BluR to negatively influence the expression of curli fibres in *E. coli*.

## Discussion

### Evolutionary origin of the *bluR-bluF-ycgZ-ymgABC* region

The *bluR-bluF-ycgZ-ymgABC* coding region from *E. coli* represents a genetic entity that acts in a common regulatory and functional context, in which the *ycgZ-ymgABC* operon provides the target genes under the direct control of the MerR-like repressor BluR and indirect influence of the BLUF-EAL protein BluF, which is able to antagonize BluR by direct interaction (Tschowri *et al*., 2009). Genomic comparisons revealed that *bluR-bluF-ycgZ-ymgAB* represents the minimal unit, which is conserved in various enterobacteria including *K. pneumoniae*, *Enterobacter* sp. (Fig. 1C) and *Citrobacter rodentium* (not shown). *ymgC*, the last gene in the *ycgZ-ymgABC* operon, as well as *ycgG* and *ymgF*, which are located right downstream of *ymgC*, are present in certain *E. coli* strains but are absent in other enteric bacteria (Fig. 1C). YcgG is another EAL-domain protein and likely c-di-GMP phosphodiesterase with unknown function and YmgF is a non-essential protein that plays a role in cell division (Karimova *et al*., 2009). It is not surprising that host-adapted pathogens like *Yersinia*, *Salmonella* and classical EHEC 0157:H7 strains do not possess the entire *bluR-bluF-ycgZ-ymgAB* unit, i.e. they have lost an apparently useless ability to sense and respond to blue light. On the upstream side, the *bluR-bluF-ycgZ-ymgABC* locus of *E. coli* is flanked by a region containing 21 genes (*ymfD*–*stfE*) from the e14 prophage. In addition, *ycgX,* which is located just next to *bluR* and is present in some but not all *E. coli* strains, has several homologues in prophages, such as *ybcV* on the DLP12 prophage or *ydfO* on the Qin prophage. The presence of diverse prophage-related genes in the direct neighbourhood of the *bluR-bluF-ycgZ-ymgAB* unit suggests that the entire region may have been acquired by phage-associated horizontal gene transfer.

### Origin and molecular function of the blue light-sensor BluF

Certain enterobacteria like *K. pneumoniae* and *Enterobacter* sp. contain two genes coding for a BLUF-EAL domain protein whereas other bacteria like most *E. coli* strains, *C. rodentium*, *B. avium* and *A. macleodii* contain just one version of this gene. Moreover, the EAL domains of the BluF-related proteins from these bacteria show different degrees of degeneration regarding key amino acids essential for phosphodiesterase activity. BluF homologues with degenerate EAL domains are usually encoded next to the gene for a MerR-like protein with pronounced similarity to BluR of *E. coli* and an operon consisting of *ycgZ-ymgAB*-related genes. On the other hand, all BluF variants, which show PDE activity like BlrP1 (KPK_2789) (Barends *et al*., 2009) or are likely to be active PDEs due to the presence of the key conserved amino acids (like Ent638_2032 from *Enterobacter* sp. 638 or BAV1542 from *B. avium*), are located in a different chromosomal context. The BluF protein from *E. coli* shows the highest degree of degeneration in comparison with other BluF homologues. Having lost the ability to bind or degrade c-di-GMP but having gained a new function as an anti-repressor of BluR (Tschowri *et al*., 2009), it represents the paradigm for a functional shift of a BLUF-EAL protein that occurred by a series of single amino acid exchanges.

In our ‘retro-evolution’ experiment, re-introducing consensus amino acids into the EAL domain of BluF from *E. coli* did not restore PDE activity nor c-di-GMP binding (Fig. S3), but rather weakened the potential of BluF to antagonize BluR. Especially BluF variants with several mutations (M4–M8) were unable to fully derepress *ycgZ* expression (Fig. 2). Nevertheless, even BluF-M8 was still able to interact with both BluR^NTD^ and BluR^CTD^ in the two-hybrid assay (Fig. 4A). In this assay, however, the interacting proteins are overproduced, meaning that BluR^NTD^ is not bound to its operator DNA, which in the natural chromosomal setting may compete with BluF for binding to BluR^NTD^.

BluF-M8 also exhibited an interesting gain of function, i.e. in comparison with wild-type BluF its interaction with the BluR paralogue MlrA and especially with the MlrA^CTD^ was improved (Fig. 4A). When the isolated EAL domains of wild-type BluF and BluF-M8 were used in the two-hybrid analysis, such a difference was not observed suggesting that interaction sites are present also in wild-type BluF, but are conformationally occluded in a way that becomes relaxed by introducing the eight point mutations present in the M8 mutant variants. Thus, a potential to interact with MlrA may have become cryptic during evolution of BluF.

All these observations taken together indicate that (i) both domains of BluF contribute in a complex way to the interactions with BluR or MlrA (that an interaction between the purified BluF^NTD^ and the two BluR domains was not observed *in vitro* (Tschowri *et al*., 2009), may have been due to non-native protein conformation of BluF^NTD^ alone), (ii) BluF is not just a defective PDE but has evolved to specifically bind to BluR, which involves contacts to both domains of BluR and (iii) BluF probably has evolved from a protein that originally had PDE activity and bound to the ligand-binding C-terminal domain of MlrA, the activator of *csgD* expression. We recently found MlrA to form a complex with the diguanylate cyclase YdaM and the PDE YciR. Within this complex, the MlrA^CTD^ interacts with the EAL-domain of YciR (S. Lindenberg and R. Hengge, unpubl. results). Similarly, BluF and especially BluF-M8 show clear interaction with MlrA^CTD^. However, this interaction does not play a physiological role anymore, but is revealed only upon overproduction of BluF. Only then it inhibits *csgD* expression (Fig. 3), possibly by inhibiting the formation of the functional YdaM–MlrA–YciR complex.

In contrast to its interaction with MlrA^CTD^ only, BluF binds to *both* domains of BluR, with interaction to the BluR^NTD^ being even stronger (Fig. 4; and Tschowri *et al*., 2009). Thus, the interaction with the DNA-binding N-terminal domain of BluR may be a novel evolutionary acquisition specific for its function as a direct antagonist that releases BluR^NTD^ from the DNA, and may involve a direct competition of BluF and operator DNA for BluR^NTD^. Consistently, the pI of the BluF^EAL^ domain is lower (4.82) than the pI of the EAL domains of the BluF homologues in *B. avium* and *A. macleodii* (5.29 and 5.28 respectively), which do not have BluR homologues, or the pI of YciR^EAL^ (6.03), which interacts with MlrA^CTD^ (Tschowri *et al*., 2009). A functionally similar case of an anti-repressor competing with operator DNA for binding to a MerR-like regulator is the CarS-CarA system in *Myxococcus xanthus*, but CarS (which is also acidic with a pI of 4.8) is not structurally related to BluF or other EAL domain proteins (Navarro-Avilés *et al*., 2007; León *et al*., 2010; Elías-Arnanz *et al*., 2011).

### Origin and molecular function of the repressor BluR

MerR-like transcriptional regulators contain an N-terminal helix–turn–helix DNA-binding region and a ligand-binding CTD. They form dimers that bind to suboptimal promoters with typically longer spacer regions (19–20 bp) and in their apo-form act as repressors. Upon binding of the inducer, they activate transcription by distorting the promoter DNA in a way that facilitates RNAP binding and allows open complex formation (summarized in Brown *et al*., 2003). The closely related MerR-like proteins BluR and MlrA of *E. coli* possibly arose by gene duplication. This view is supported not only by the high amino acid identity (49% over the entire same length of the two domains of each of the two proteins; Fig. S2), but also by the observation that BluR still exhibits residual binding to the MlrA-regulated *csgD* promoter and, upon overproduction, can directly influence curli expression (Fig. 6). A similar evolutionary origin has also been suggested for two other closely related MerR-like transcription factors, CueR and GolS in *Salmonella* (Pérez-Audero *et al*., 2010).

By evolving further BluR then may have acquired specific properties that distinguish it from MlrA and other MerR-like regulators. Most importantly, BluR seems to act as a repressor only. This is consistent with its binding site in the *ycgZ* promoter, which includes the −35 hexamer and part of the spacer region (Fig. 5). Also at its non-physiological target, the *csgD* promoter, overexpressed BluR inactivates expression, although it replaces an activator, i.e. MlrA, in this situation. In view of the strong similarity of BluR and MlrA (Fig. S2), it would be interesting to clarify, how and why BluR lost its ability to activate gene expression and now acts as a repressor only. One reason may be that BluR has lost the ability to bind a ligand, which typically accounts for the specificity of a MerR-like regulator, and therefore cannot switch to an activating conformation anymore. MlrA interacts with the diguanylate cyclase YdaM (S. Lindenberg and R. Hengge, unpubl. results). MlrA^CTD^ contains a RxxD motifs, i.e. a signature involved in c-di-GMP binding in several types of c-di-GMP effector proteins (Schirmer and Jenal, 2009), but none of the cysteines, which in metal-responding MerR-like proteins, e.g. MerR, ZntR and CueR, are involved in ligand binding to the CTD (Helmann *et al*., 1990; Brown *et al*., 2003; Changela *et al*., 2003). In comparison, BluR contains neither the RxxD motif nor any cysteines in its CTD. These observations suggest that during evolution BluR may have lost its ability to bind a specific ligand and therefore cannot assume an activating conformation. Instead, BluR has adapted to interact with BluF, which allows a derepression of target genes by a release of BluR from the operator region.

### A potential evolutionary scenario: emergence of the BluF–BluR blue light signalling pathway generated a link between CsgD/curli biosynthesis and the Rcs pathway

The BluF–BluR system provides a unique opportunity to study the course of evolution not just theoretically but also experimentally. Comparative genome and sequence analyses performed in this study suggest that the BLUF-EAL protein BluF of *E. coli*, which acts as a direct antagonist for the repressor and MlrA paralogue BluR, originated from a blue light-regulated PDE not functionally associated with a MerR-like protein. This situation is still found for instance in *B. avium* and *A. macleodii*, which do not possess any BluR homologues but a BluF variant that contains all key residues required for a c-di-GMP-hydrolysing PDE (Figs 1C and S1). Thus, the two functions of BluF proteins as an active PDE and an anti-repressor represent two evolutionary extremes. The ‘missing link’ between these two versions of BluF would be an active PDE that acted as a direct antagonist to MlrA and in doing so acquired the capability to control curli expression via the MlrA target gene *csgD*. This situation could be simulated experimentally in *E. coli* by re-introducing certain PDE-typical residues into BluF, which – despite not yielding enzymatic activity – enhanced a residual or cryptic interaction between BluF and MlrA^CTD^ (Fig. 4A). A similar situation may naturally exist in *K. pneumoniae*, which, besides a BluF homologue with a degenerate EAL domain that is genetically associated with a BluR orthologue (Fig. 1C), also possesses the enzymatically active BluF variant (BlrP1) (Barends *et al*., 2009) and a MlrA orthologue.

In *E. coli*, MlrA is part of a complex with the DGC YdaM and the PDE YciR in which MlrA^CTD^ directly contacts the EAL domain of YciR (S. Lindenberg and R. Hengge, unpubl. data). One may speculate that competition for MlrA^CTD^ between the EAL domains of YciR and BluF could have provided the opportunity or even a selective pressure for a duplication of the *mlrA* gene. During the co-evolution of *bluF* with the new copy of *mlrA*, BluF may have lost its ability to bind and degrade c-di-GMP, whereas the extra copy of *mlrA* evolved into *bluR* with its gene product becoming a repressor for a new target, i.e. the *ycgZ* promoter. The observation that BluF still shows residual binding to MlrA (Figs 3 and 4) and BluR still has low affinity for the *csgD* promoter region (Fig. 6) suggests that evolution of BluF/BluR occurred rather recently in enteric bacteria.

**Fig. 7:**
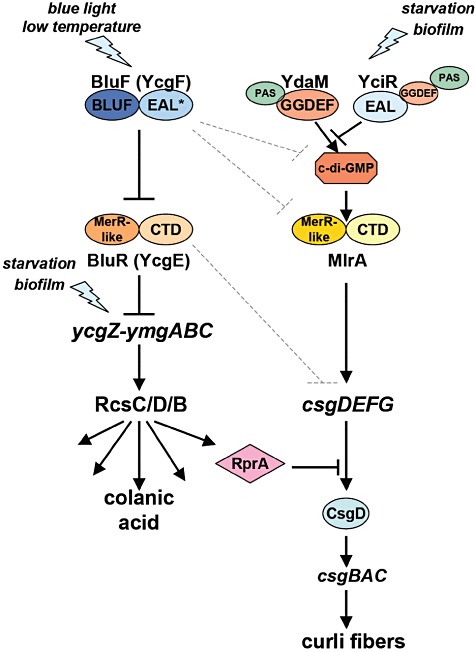
Graphical summary of the roles of the BluF/BluR and YdaM/YciR/MlrA systems in the control of expression of CsgD and curli fibres. In *E. coli* K-12, transcription of *csgD*, which encodes a key biofilm regulator that activates the curli *csgBA* operon, is controlled by the YdaM/MlrA/YciR pathway (Weber *et al*., 2006). On the other hand, the BluF–BluR-YcgZ/YmgA/YmgB-RcsC/RcsD/RcsB pathway – via the small RNA RprA – controls abundance and translation of *csgD* mRNA, and therefore also the expression of CsgD (Tschowri *et al*., 2009; Mika *et al*., 2012). The small proteins YmgB and, to a lesser extent, YmgA activate the Rcs phosphorelay system (Tschowri *et al*., 2009), which control the expression of numerous genes involved in stress responses and biofilm formation (Majdalani and Gottesman, 2005). Residual ‘cross-talk’, i.e. the potential for specific interactions between components of the two pathways (as revealed in this study), is indicated by hatched lines. These effects are likely to reflects the evolutionary origins of the paralogues BluR and MlrA and their interaction partners as outlined in the main text.

So what were the physiological consequences of this evolutionary scenario? The physiological link of all potential evolutionary intermediates of BluF – irrespective of acting as a PDE or directly antagonizing MlrA at the *csgD* promoter or BluR at the *ycgZ* promoter – was their ability to inhibit the expression of curli fibres as a response to blue light (Fig. 7). However, the actual mode of inhibition changed. Thus, BluF may have ‘started’ as simple PDE that would just maintain low cellular c-di-GMP levels. Then it may have evolved into a factor that via interaction with MlrA directly and locally antagonized *csgD* transcription. Finally, it became an indirect inhibitor of *csgD* expression by antagonizing BluR and thereby activating the Ymg/Rcs pathway, which stimulates expression of the small RNA RprA that downregulates *csgD* at the mRNA level (Tschowri *et al*., 2009; Mika *et al*., 2012). Overall, BluF thus remained an inhibitor of curli expression, but its more complex influence via the Ymg/Rcs pathway now allowed it to integrate new environmental signals such as low temperature (Tschowri *et al*., 2009) and to significantly expand its target range to now include the Rcs regulon (Majdalani and Gottesman, 2005). As a consequence, the recently evolved BluF–BluR–Ymg–Rcs–RprA–CsgD pathway established a link between early (CsgD-dependent) and late (Rcs-modulated) events in stationary phase and during biofilm formation.

## Experimental procedures

### Bacterial strains and growth conditions

All strains used in this study are derivatives of the *E. coli* K-12 strain W3110 (Hayashi *et al*., 2006) containing a *lac(I-A)::scar* deletion. The *bluR::cat*, *bluF::kan, ymgB::kan* and *yhjH::kan* were previously described (Pesavento *et al*., 2008; Tschowri *et al*., 2009) and represent deletion–insertion mutations generated by one-step inactivation according to Datsenko and Wanner (2000). *ymgA::kan* was similarly constructed (for oligonucleotide primers, see Table S1). Mutations were transferred by P1 transduction (Miller, 1972).

Cells were grown in LB medium (Miller, 1972) under aeration at 28°C or 37°C. Ampicillin and IPTG were added as recommended (Miller, 1972). Growth was monitored by measuring the optical density at 578 nm (OD_578_).

### Construction of plasmids and *lacZ* reporter fusions

The primers used for plasmid constructions are listed in Table S1. Point mutations in the *ycgZ* promoter region and *bluF* were generated using a four-primer/two-step PCR protocol (Germer *et al*., 2001) and primers listed in Table S1. Mutated *bluF* variants were cloned into a pQE30Xa (Qiagen) derivative, which also carries the *lacl^q^* gene inserted at the XbaI site. The mutations introduced into BluF were as follows: M2: I193L+Q195R, M4: M2+M362E+A365E, M5: M4+T247N, M6: M5+H177Q, M7: M6+H306D and M8: M7+S328D. The same external primers used for cloning of wild-type BluF into pQE30Xa were utilized exactly as described in Tschowri *et al*. (2009).

In order to construct *lacZ* fusions to different *ycgZ* promoter region variants containing mutations in the BluR binding sites, the appropriate PCR fragments were cloned into the *lacZ* fusion vector pJL28 as previously described (Tschowri *et al*., 2009). The resulting plasmids served as templates for the isolation of mutated *ycgZ* promoter DNA fragments used in the electrophoretic mobility shift assays. All *lacZ* reporter fusions were transferred to the *att*(lambda) location of the chromosome via phage λRS45 (Simons *et al*., 1987) and tested for single lysogeny by PCR (Powell *et al*., 1994). The single copy *csgB::lacZ* fusion was constructed in the same way and was described earlier (Weber *et al*., 2006).

pCAB18 (Barembruch and Hengge, 2007) is a *tac* promoter expression plasmid based on the low-copy-number vector pACYC184 (Chang and Cohen, 1978) and was used for cloning of *bluR* (using oligonucleotides listed in Table S1) and *yhjH*[as described in Pesavento *et al*. (2008)].

For *in vitro* protein interaction analysis, S-tagged MlrA was generated using pETDuet (Merck, previously Novagen) and primers listed in Table S1. Tag-free BluR used for gel retardation experiments was expressed with an N-terminal intein tag from pTYB12 (New England Biolabs) as described in the Supplement for (Tschowri *et al*., 2009).

For *in vivo* interaction assays the BacterioMatch II Two-Hybrid System (Stratagene, Agilent Technologies) was used. The relevant proteins were expressed as C-terminal fusions either to the lambda cI repressor (from pBT) or to the N-terminal domain of the alpha subunit of *E. coli* RNAP (from pTRG) (Dove and Hochschild, 2004).

### Determination of transcriptional start sites by primer extension

RNA preparation and primer extension was performed as described (Bouvier *et al*., 2008) with minor alterations. To determine the transcriptional start sites of *bluR* and *bluF* cells were grown in LB at 37°C until an OD_578_ of 0.7 and then transferred to 16°C prior to sample collection from the overnight culture. To specify the transcriptional start of the *ycgZ-ymgABC* operon, RNA samples were taken from cells grown overnight at 37°C. Wild-type MC4100, its *bluR::cat*, *bluF::kan* and *ycgZymgAB::cat* mutant derivatives and MC4100 containing a derivative of pJL28 (see above) containing DNA fragments comprising the *bluR*, *bluF* or *ycgZ* promoter regions were used for total RNA isolation with the SV RNA Isolation Kit (Promega).

To detect the transcriptional start sites by primer extension, primers listed in Table S1 were labelled with [γ-^32^P]-ATP and T4 PNK (Fermentas) and incubated with 10 µg of total RNA and 200 U of SUPERSCRIPT II (Invitrogen) at 45°C for 60 min. The reaction was stopped by incubation at 70°C for 15 min. A DNA sequence ladder was generated with the same labelled primer using the CycleReader DNA Sequencing Kit (Fermentas). For reaction termination, STOP solution supplied with the kit was added to the primer extension samples, which were run on 6% polyacrylamid 7 M urea sequencing gels after heating to 90°C for 3 min. The gels were dried before being analysed using a FLA-2000G Imager (Fuji Photo Film, Japan).

### Protein overexpression and purification

N-terminally His-tagged BluF and its mutated variants were purified after overexpression from pQE30Xa-derived plasmids. After transformation cells were grown at 37°C in LB/ampicillin (100 µg ml^−1^) to an OD_578_ of 0.7 before the addition of 1 mM IPTG and cultures were transferred to 16°C for overnight growth. Cells were harvested and proteins were purified according to a standard protocol (QIA expressionist manual; Qiagen) as described (Tschowri *et al*., 2009). Overexpression and purification of a tag-free BluR from pTYB12 was previously described in the Supplement to Tschowri *et al*. (2009). To overexpress S-tagged MlrA, ER2566 cells carrying pETDuet-encoded MlrA were grown in LB/ampicillin at 37°C to an OD_578_ of 0.5–0.7, when IPTG (100 µM) was added and incubation continued overnight at 16°C. pQE60 (Qiagen) encoded C-terminally His6-tagged YhjH (Pesavento *et al*., 2008) was purified from cells grown at 37°C to an OD_578_ of 0.8 prior to addition of IPTG (0.5 mM) and subsequent culture incubation at 25°C for 4 h. The diguanylate cyclase PleD* was purified exactly as described (Paul *et al*., 2004).

### Protein–DNA interaction assays

Electrophoretic mobility shift assays (EMSA) were performed in bandshift buffer (10 mM Tris-HCl at pH 7.5, 1 mM EDTA, 5% glycerol, 10 mM NaCl and 1 mM MgCl_2_) in 20 µl of reaction mixtures containing 1.42 µM or indicated amounts of tag-free BluR, 107 fmol of DNA fragments comprising the promoter region of *ycgZ* (p*ycgZ*, 158 bp) or *csgD* (p*csgD*, 198 bp) as well as 1 µg of poly[d(I-C)] (Roche) as non-specific competitor DNA. The DNA fragments were generated using primers listed in Table S1 (see below) and purified by gel electrophoresis with subsequent gel extraction. Reaction mixtures were incubated for 30 min at room temperature and then run on a 5% polyacrylamid gel in 0.5× TBE buffer followed by ethidium bromide staining.

To identify BluR binding sites in the *ycgZ* promoter region DNase I footprint analysis was performed as described (Mika and Hengge, 2005) with minor alterations. A DIG-labelled DNA fragment (176 bp) containing the *ycgZ* promoter region was generated by PCR using primers listed in Table S1. Complex formation between the DIG-labelled DNA fragment (260 fmol) and increasing amounts of BluR (0–3.94 µM) was performed in 20 µl of reaction mixtures for 60 min at room temperature and otherwise as described above for the EMSA. A DNA sequence ladder was generated with the CycleReader DNA Sequencing Kit (Fermentas) and the same DIG-labelled primer as used for generation of the DNA fragment (Table S1).

### *In vitro* protein–protein interaction assay, SDS polyacrylamide gel electrophoresis and immunoblot detection of proteins

*In vitro* interaction assays were performed by affinity chromatography (‘pull-down’ assays) on S-protein agarose (Merck, previously Novagen) using extracts of cells expressing plasmid-encoded S-tagged MlrA or His6-tagged BluF. Cells were grown as described above and after harvesting resuspended in binding buffer (20 mM Tris-HCl PH 7.5, 150 mM NaCl, 5 mM MgCl_2_) in 100-fold concentration. Cell lysis was obtained by passage through a French Press and after centrifugation at 15.000 r.p.m. for 40 min the soluble protein fraction was analysed by SDS-PAGE. According to relative protein concentrations (as observed by the SDS-PAGE analysis) 100 µl of the cell extract containing S-tagged MlrA was mixed with 900 µl of extract of cells overexpressing His6-tagged BluF or the same amount of a control extract obtained with cells containing the empty vector only. Sixty microlitres of S-protein agarose slurry was added and the mixture was incubated for 30 min at room temperature. After washing four times with 500 µl of binding buffer samples were eluted with 40 µl of 3 M MgCl_2_.

Eluates or whole-cell extracts were subject to SDS-PAGE and immunoblot analysis as described previously (Lange and Hengge-Aronis, 1994). To determine cellular levels of CsgD, 10 µg of cellular protein was applied per lane. Polyclonal sera against CsgD (custom-made by Pineda-Antikörper-Service, Berlin) or a monoclonal anti-His-tag antibody (Sigma) goat anti-rabbit and anti-mouse IgG alkaline phosphatase conjugate (Sigma) and a chromogenic substrate (BCIP/NBT; Boehringer Mannheim) were used.

### Two-hybrid analysis for testing protein–protein interactions *in vivo*

To test protein–protein interaction *in vivo*, the Bacterio-Match two-hybrid system (Agilent Technologies) was used according to the manufacturer's protocol. Proteins to be tested for interaction are fused to the N-terminal DNA-binding domain of the lambda cI repressor (expressed from pBT) and to the N-terminal domain of the bacterial RNA polymerase alpha subunit (expressed from pTRG) (Dove and Hochschild, 2004). When interaction occurs, expression of the *HIS3* gene (originally from *Saccharomyces cerevisiae*) is sufficiently activated in the *E. coli* reporter strain (a derivative of XL1-Blue MRF′) to allow growth on selective medium (containing 5 mM of the His3 inhibitor 3-Amino-1,2,4-triazole, 3-AT). Growth on selective plates was monitored by counting numbers of co-transformants directly plated on selective plates (expressed in relation to numbers directly obtained on non-selective plates) as well as by growth of co-transformants obtained on non-selective plates that were restreaked in patches on selective plates. Occasionally observed reduced growth on non-selective plates indicates a detrimental effect of overproduction of one of the partner proteins. If such reduced growth occurs with only one protein overproduced, it usually is improved when an interacting partner protein is expressed from the other vector.

### Determination of c-di-GMP binding and phosphodiesterase activity

*In vitro* synthesis of radiolabelled c-di-GMP from [α-^32^P]-GTP by the purified diguanylate cyclase PleD* and purification of c-di-GMP was performed as described (Paul *et al*., 2004; Weber *et al*., 2006). Binding of radiolabelled c-di-GMP to purified proteins *in vitro* was detected by UV cross-linking according to Christen *et al*. (2005). Phosphodiesterase activity was tested with purified BluF and its mutated variants under blue light conditions as described in Tschowri *et al*. (2009) as well as with C-terminally His6-tagged YhjH (Pesavento *et al*., 2008) using radiolabelled c-di-GMP as a substrate. The products were analysed by thin-layer chromatography according to Weber *et al*. (2006).

### Determination of β-galactosidase activity

β-Galactosidase activity was assayed by use of *o*-nitrophenyl-β-d-galactopyranoside (ONPG) as a substrate and is reported as µmol of *o*-nitrophenol per min per mg of cellular protein (Miller, 1972). Experiments showing the expression of *lacZ* fusions as single-value data were performed at least three times, with the average of these three independent measurements being shown.

### Bacterial motility assay

Motility was tested on soft agar plates containing 0.5% bacto-tryptone, 0.5% NaCl and 0.3% agar. Three microlitres of an overnight culture (adjusted to an OD_578_ of 4.0 in its own supernatant) was inoculated into the plates and cells were allowed to grow and swim for 5 h at 28°C.

### DNA and protein sequence analyses

The blast program was used to search the NCBI data library (Altschul *et al*., 1997). Multiple alignments of EAL domains and of MerR-like proteins were generated by clustal w (Larkin *et al*., 2007). Comparative genome analysis were performed using EcoCyc (Keseler *et al*., 2011).
