# Supplementary material for: Molecular function and potential evolution of the biofilm-modulating blue light-signalling pathway of Escherichia coli
Source: Mol Microbiol. 2012 Jul 12;85(5):893–906. doi: 10.1111/j.1365-2958.2012.08147.x (PMC3509220; doi:10.1111/j.1365-2958.2012.08147.x)
Supplement: Supplementary file 1 [file mmi0085-0893-SD1.pdf]

Supplementary Material for:

Molecular function and potential evolution of the biofilm-modulating blue light-signaling pathway of *Escherichia coli*

Natalia Tschowri, Sandra Lindenberg and Regine Hengge

Institut für Biologie, Mikrobiologie, Freie Universität Berlin, 14195 Berlin, Germany

Contents:

Figure S1.
EAL-domain sequence alignment of various BluF homologs in comparison to the EAL-domain of the active phosphodiesterase (PDE) YahA.

Figure S2.
Sequence comparison of BluR with other MerR-like proteins from *E.coli*.

Figure S3. Restoration of consensus amino acids in the EAL domain of BluF does not reconstitute PDE activity.

Figure S4.
Two-hybrid analysis to detect *in-vivo* interactions between BluF-WT and BluF-M8 and MlrA as well as the single domains of MlrA and BluR.

Table S1.
Oligonucleotides used in the present study

Supplementary References

Supplementary Figures

```
YahA            -----------HEFKPWIQPVFCAQTGVLTGCEVLVRWEHPQTGIIPPDQFIPLAESSGL 168
BAV1542         TDRSDIAQNDDPPYQFALQPIVNPMRRDISSFEFLIRGRNGGS---PEQFFSSYPAEH-- 220
KPK_2789        -------NLTDQPCQFALQAIVEPAKRRVSSFEALIRSPTGGS---PVEMFAAIAAED-- 210
Ent638_2032     -----LISN--QICQFAFQPIVDPSEGKITSLEALIRGNDGGS---PEHFFNALDPDR-- 213
KPK_3794        -------IDNTESWSFAFQPIVDPFACEIISWEALLRTPDGQS---PGAYFAGLAGDD-- 213
Ent638_1757     -------IAKGADCSFAFQPIVDPLTQQVVSWEALLRTPSGGS---PAVYFESLTGNA-- 224
BluF            -------LSPTINDHFAFHPIVDPLSRRIIAFEAIVQKNEDSP---SAIAVGQRKDGE-- 213
                                  *               * *

YahA            IVIMTRQLMKQTADILMPVKHLLPDNFHIGINVSAGCFLAAG-FEKECLNLVNKLGNDKI 227
BAV1542         ---RYRLDIESKALAFRLAHQIGQQHIKVAVNLFPMSLMTVPDAVDKLVAHIEASGLSPT 277
KPK_2789        ---RYRFDLESKAFAFSLAARLPLGKQQLAVNLLPGSLYNHPDAVGWLMDNLLAAGLRPE 267
Ent638_2032     ---IYEIDLQTKAYAFALADKIGIGDHKIAINLLPMSLVNVPGAVEFLVESIAKHHLLPE 270
KPK_3794        ---IYLADLHSKRVALSLAGKLGLRNKALSINLLPMTLVKAPNAVAFLLDEISRNDLIPE 270
Ent638_1757     ---IYESDLKSKQVALSMASALGLEGQTLSLNLLPMTLVNVPNAVDFLLKEIAANGLVPE 281
BluF            ---IYTADLKSKALAFTMAHALELGDKMISINLLPMTLVNEPDAVSFLLNEIKANALVPE 270
                                                    *

YahA            KLVLELTERNPIPVTPEARAIFDSLHQHNITFALDDFGTGYATYRYLQAFPVDFIKIDKS 287
BAV1542         QVLVEITEQEAISCLRSFGNAIKRLRAFGVGVAIDDFGSGFAGLSLLAEFQPDKLKIDRR 337
KPK_2789        QVLIEVTETEVISCFDQFRKVLKALRVAGMKLAIDDFGAGYSGLSLLTRFQPDKIKVDAE 327
Ent638_2032     QVVVEVTENEMISGFNQFNSAIKQLRAAGIGLAIDDFGSGYAGLSLLTKFQPDKIKIDRE 330
KPK_3794        QIIVEFTEREVISRMADFTDAVRKLKGAGINLAIDHFGAGFAGLSLLAQYQPDRIKIDHE 330
Ent638_1757     QIIIEFTESEVISRLTEFKGAVRQLKSAGISVAIDHFGAGFAGLLLLAQFQPDRIKINRE 341
BluF            QIIVEFTESEVISRFDEFAEAIKSLKAAGISVAIDHFGAGFAGLLLLSRFQPDRIKISQE 330
                                                   *                     *

YahA            FVQMASVDEISGHIVDNIVELARKPGLSIVAEGVETQEQADLMIGKGVHFLQGYLYSPPV 347
BAV1542         IIQDIHTDGPRQAIVLAIVQVCTAMGITPVAEGVESIDEWCWLQAAGVERFQGYLFAKPA 397
KPK_2789        LVRDIHISGTKQAIVASVVRCCEDLGITVVAEGVETIEEWCWLQSVGIRLFQGFLFSRPC 387
Ent638_2032     IITNIHRSGPKQAIVKSIISCCTDMEITLVAEGIEQIDEWCWLESAGIKRFQGFLFARPQ 390
KPK_3794        LIRNIHQDGPRQSIVQAIIKCCTSLEIAVSAVGVERAEEWMWLESAGISQFQGNLFAGAR 390
Ent638_1757     LVADVHKSGPRQAIVQAIIKCCASLEIQFCAVGVEKAEEWMWLESAGISEFQGHLFASPK 401
BluF            LITNVHKSGPRQAIIQAIIKCCTSLEIQVSAMGVATPEEWMWLESAGIEMFQGDLFAKAK 390
                                               *  *
```

■ c-di-GMP-binding ■ Mg^{2+}-binding ■ catalysis

Figure S1. **EAL-domain sequence alignment of various BluF homologs in comparison to the EAL-domain of the active phosphodiesterase (PDE) YahA.** The alignment was generated using CLUSTAL W (Larkin *et al*., 2007) and further adjusted manually to highlight key amino acid positions, which have been assigned to conserved amino acids in active PDEs required for c-di-GMP binding (red), Mg^{2+}-binding (blue) and catalysis (green) using RocR from *Pseudomonas aeruginosa* as a model system (Rao *et al*., 2008). YahA from *E. coli* was experimentally demonstrated to be active as a c-di-GMP specific phosphodiesterase by (Schmidt *et al*., 2005).

The EAL domain sequences of BluF homologs from *Bordetella avium* 197N (BAV1542), *Klebsiella pneumoniae* 342 (KPK_2789 and KPK_3794), *Enterobacter sp*. 638 (Ent638_2032 and Ent638_1757) and for *E. coli* K-12 (BluF) are depicted. KPK_3794 and Ent638_1757 contain a partially degenerate EAL-domain and are encoded next to the genes for BluR homologs (see Fig. 1C). KPK_2789 (BlrP1), which is encoded independently of BluR, contains a conserved EAL-domain and was shown to act as a blue-light regulated PDE (Barends *et al*., 2009). Due to amino acid sequence, Ent638_2032 and BAV1542 very likely represent active PDEs and are not associated with BluR in terms of their genetic location. BluF from *E.coli*, which is encoded next the gene for BluR lacks all four amino acids involved in c-di-GMP binding, one of eight amino acids involved in Mg^{2+}-binding and an essential catalytic glutamate residue. It represents the most degenerated BluF variant and was shown to be unable to bind or degrade c-di-GMP, but to act as anti-repressor protein (Tschowri *et al*., 2009). Key amino acids essential for PDE activity that were re-introduced into BluF (resulting in BluF M2-M8 variants; see Figs. 2, 3, 4) are indicated by asterisks.

```
                      helix-turn-helix region
BluR   --------MAYYSIGDVAERCGINPVTLRAWQRRYGLLKPQRSEGGHRLFDEEDIQRIEE 52
MlrA   --------MALYTIGEVALLCDINPVTLRAWQRRYGLLKPQRTDGGHRLFNDADIDRIRE 52
SoxR   MEKKLPRIKALLTPGEVAKRSGVAVSALHFYESKGLITS-IRNSGNQRRYKRDVLRYVAI 59
CueR   -----------MNISDVAKITGLTSKAIRFYEEKGLVTPPMRSENGYRTYTQQHLNELTL 49
ZntR   ----------MYRIGELAKMAEVTPDTIRYYEKQQMMEHEVRTEGGFRLYTESDLQRLKF 50

BluR   IKRWISNGVPVGKVKALLETTSQDTEDDWSRLQEEMMSILRMANPAKLRARIISLGREYP 112
MlrA   IKRWIDNGVQVSKVKMLLSNENVDVQNGWRDQQETLLTYLQSGNLHSLRTWIKERGQDYP 112
SoxR   IKIAQRIGIPLATIGEAFG-----VLPEGHTLSAKEWKQLSSQWREELDRRIHTLVA--- 111
CueR   LRQARQVGFNLEESGELVN-----LFNDPQRHSADVKR-RTLEKVAEIERHIEELQS--- 100
ZntR   IRHARQLGFSLESIRELLS-----IRIDPEHHTCQESKGIVQERLQEVEARIAELQS--- 102

BluR   VDQLINHVYLPVRQRLVLDHNTSRIMSSMFDGALIEYAATSLFEMRRKPGKEAILMAWNV 172
MlrA   AQTLTTHLFIPLRRRLQCQQPTLQALLAILDGVLINYIAICLASARKKQGKDALVVGWNI 172
SoxR   ----------LRDELDGCIGCGCLSRSDCPLRNPGDRLGEEGTGARLLEDEQN------- 154
CueR   ----------MRDQLLALANACPG--DDSADCPIIENLS-GCCHHRAG------------ 135
ZntR   ----------MQRSLQRLNDACCGTAHSSVYCSILEALEQGASGVKSGC----------- 141

BluR   EERARLWLEAWRLSLSGWHISVLADPIESPRPELFPTQTLIVWTGMAPTRRQNELLQHWG 232
MlrA   QDTTRLWLEGWIASQQGWRIDVLAHSLNQLRPELFEGRTLLVWCGENRTSAQQQQLTSWQ 232
SoxR   ------------------------------------------------------------
CueR   ------------------------------------------------------------
ZntR   ------------------------------------------------------------

BluR   EQGYKVIFHAP 243
MlrA   EQGHDIFPLGI 243
SoxR   -----------
CueR   -----------
ZntR   -----------
```

Figure S2. **Sequence comparison of BluR with other MerR-like proteins from *E. coli*.** Alignments were performed using CLUSTAL W (Larkin *et al*., 2007). Residues identical to BluR were manually coloured in red. The protein sequences for BluR (Tschowri *et al*., 2009), MlrA (Brown *et al*., 2001), SoxR (Nunoshiba *et al*., 1992), CueR (Outten *et al*., 2000), and ZntR (Brocklehurst *et al*., 1999) are shown. The conserved DNA binding helix-turn-helix region is indicated above the alignment according to (Brown *et al*., 2001). The N-terminal domains were determined using SMART (Letunic *et al*., 2012) and are highlighted in gray. Based on the analysis using CLUSTAL W, BluR shows 49% identity to MlrA, 12% identity to SoxR, 17% to CueR and 14% to ZntR.

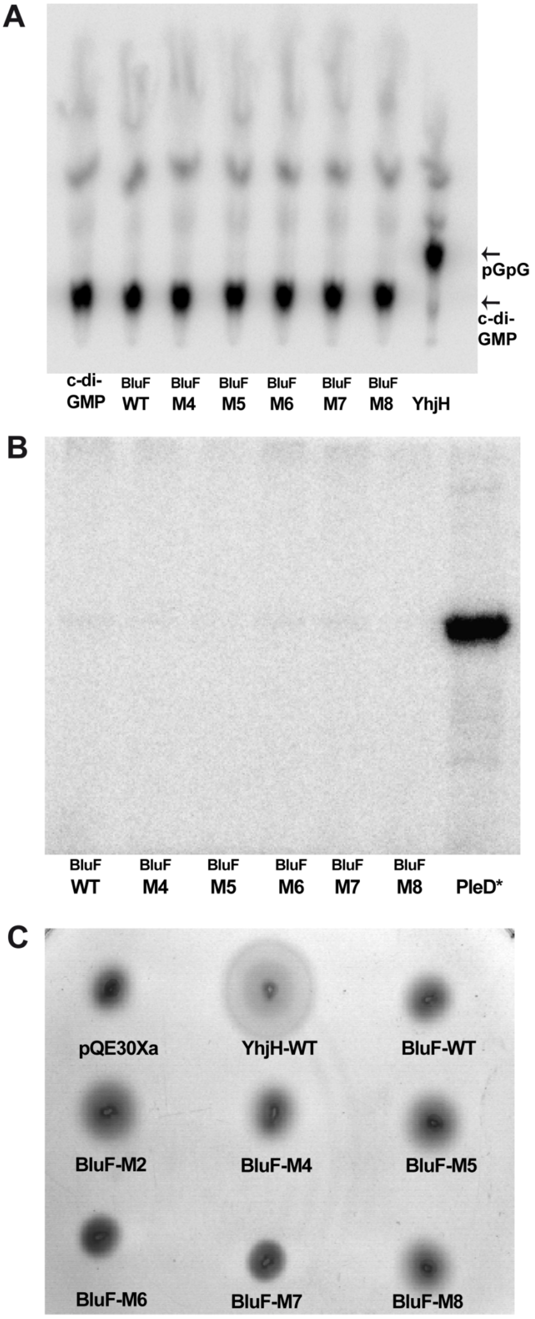

Figure S3. Restoration of consensus amino acids in the EAL domain of BluF does not reconstitute PDE activity. (A) Phosphodiesterase assay with radiolabeled c-di-GMP. Wildtype BluF (WT), the mutant variants BluF-M4 (I193L+Q195R+M362E+A365E), BluF-M5 (M4+T247N), BluF-M6 (M5+H177Q), BluF-M7 (M6+H306D), BluF-M8 (M7+S328D) and the known c-di-GMP phosphodiesterase YhjH were purified and tested for phosphodiesterase activity performed under blue-light conditions according to (Tschowri *et al.*, 2009). (B) Detection of c-di-GMP binding by UV-crosslinking. The same purified BluF proteins as used in (A) as well as the mutationally activated diguanylate cyclase PleD* were incubated with radiolabeled c-di-GMP and UV-crosslinked. (C) Motility of a W3110 *yhjH::kan* mutant carrying derivatives of pQE30Xa containing wild-type or mutated *bluF* variants as in (A) including the M2 variant ($BluF^{I193L+Q195R}$). *YhjH* was expressed from the low copy number p(*tac*) vector pCAB18. Cells were incubated on motility plates containing 100μg/ml ampicillin at 28°C for 5 hours.

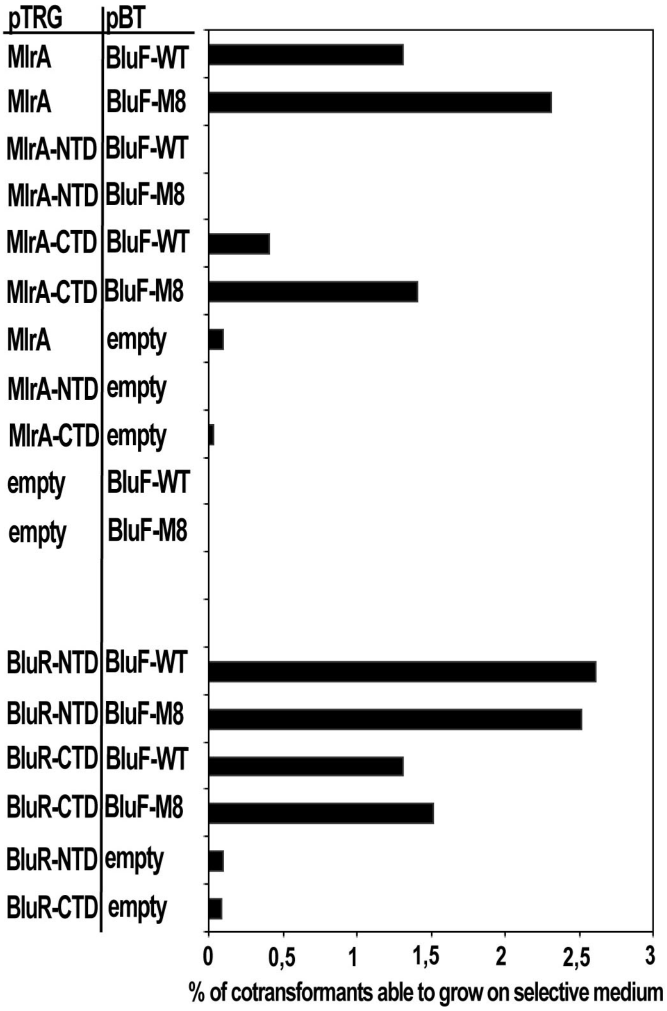

Figure S4. Two-hybrid analysis to detect *in-vivo* interaction between BluF-WT and BluF-M8 and MlrA as well as the single domains of MlrA and BluR. Using the Bacterio-Match two-hybrid system, reporter cells were co-transformed with a vector control and/or derivatives of the pBT and pTRG plasmids. Wildtype (WT) BluF and the M8 mutant version (I193L+Q195R+M362E+A365E+T247N+H177Q+H306D+S328D) were expressed as hybrid proteins fused to cI-NTD on pBT. MlrA as well as the N-terminal (NTD) and C-terminal (CTD) domains of MlrA and BluR were synthetised from pTRG as fusions to RNAP alpha-NTD. Interaction was detected by growth of cotransformants in the presence of 5 mM His3 inhibitor 3-AT (selective medium) at 37°C for 24 hours following incubation at 28°C for 48 hours. Numbers of cotransformants able to grow on selective screening medium normalized to numbers on non-selective medium are shown.

Supplementary Table[1]

Table S1. Oligonucleotides used in the present study

I. Primers for generating knockout mutations by one-step inactivation

ΔymgA::kan	5’- ATGAAGTCGTTATGAAGACATCTGATAATGAACGTATAA AATATGAAATTGTGTAGGCTGGAGCTGCTTC -3’<br>5’- TGTATTCTGTTTATTTTCTTACCATTGAACATACTTTTATT GTCAGCTGTATTCCGGGGATCCGTCGACC -3’

II. Primers for generating DNA fragments used for electrophoretic mobility assays

csgD	5’- CACCGAAATATTTTTTATATGC -3’<br>5’- CAATCTAGCCATTACAAATCTTA -3’
ycgZ	5’- ATGCATTAGCACTAATTGCA -3’<br>5’- GTGATTGCTCCCGCAGAATC -3’

III. Primers for generating a *ycgZ* promoter-containing DNA fragment for DNase I footprint assays

*ycgZ*p-fw<br>*ycgZ*p-rev	5’- DIG-GTATAGATTGTCAGTTAAATGATGC -3’<br>5’- GATGCATGCTACGCCTCTG -3’

IV. Primers for determining transcriptional start sites by primer extension

bluR	5’- GCCACAATTCCTCCTTGCGGTC -3’
bluF	5’- CGTCGTCACGTATATGGCTACG -3’
ycgZ	5’- GTGATTGCTCCCGCAGAATC -3’

V. Primers for cloning and mutagenizing the *ycgZ* promoter on pJL28

y*cgZ*-EcoRI-fw<br>y*cgZ*-HindIII-rev	5’- CG***GAATTC***GACAGGTTCGTCGTCACGTATATGGC -3’<br>5’- CGTGAC***AAGCTT***GAGTGATTGCTCCCGCAGAATC -3’
C-39A, T-38G	5’- ATCATT**AG**GTACACATATTTC -3’<br>5’- GAAATATGTGTAC**CT**AATGAT -3’
C-39A, T-38G, G-37A	5’- ATCATT**AGA**TACACATATTTC -3’<br>5’- GAAATATGTGTA**TCT**AATGAT -3’
G-24A, C-21A	5’- CACATATTTC**A**TA**A**AAGTTTGC -3’<br>5’- GCAAACTT**T**TA**T**GAAATATGTG -3’

[1]Nucleotides in **bold** indicate mutations introduced, nucleotides in ***bold italics*** indicate restriction sites.

T-23C, A-22G, A-20G	5’- CACATATTTCG**C**G**C**G**AG**TTTGC -3’<br>5’- GCAAACT**C**G**C**G**C**GAAATATGTG -3’
G-24A, T-23C, A-22G, C-21A, A-20G	5’- CACATATTTC**ACGAG**AGTTTGC -3’<br>5’- GCAAACT**CTCGT**GAAATATGTG -3’

VI. Primers used for cloning of *bluR* into pCAB18

bluR-EcoRI-fw	5’- CG***GAATTC***AGGAGGTACTGAGGTGGCTTATTACAGCATTGGTG -3’
bluR-HindIII-rev	5’- CGTGAC***AAGCTT***CGCAACGTTCAGCAACATCACC -3’

VII. Primers used for cloning into pBT

bluF (WT) and *bluF* (M8)	5’- CG***GAATTC***CCTTACCACCCTTATTTATC -3’<br>5’- CG***CTCGAG***TCATTTTTTCTCTGGCCACGCTATGG -3’
bluF-NTD	5’- CG***GAATTC***CCTTACCACCCTTATTTATC -3’<br>5’- CGC***CTCGAG***TCAGGTTGATTGTTCGGTTGCAAGGA -3’
bluF-CTD (WT) and *bluF*-CTD (M8)	5’- CGC***GAATTC***CCGCCTGCACGAGCGAGATGA -3’<br>5’- CG***CTCGAG***TCATTTTTTCTCTGGCCACGCTATGG -3’
rcsD	5’- CG***GAATTC***CCGCCATTTCTCCAGCCGC -3’<br>5’- CG***CTCGAG***TCACAGCAAGCTCTTGACATAAC -3’

VIII. Primers used for cloning into pTRG

mlrA	5’- GTAC***GAATTC***AGATGGCGCTTTACACAATTGGTG -3’<br>5’- GCTA***CTCGAG***TTAAATGCCGAGTGGGAAAATATCATGGCC -3’
mlrA-NTD	5’- GTAC***GAATTC***AGATGGCGCTTTACACAATTGGTG -3’<br>5’- GCTA***CTCGAG***CCACGTTCGCAGGCTATGTAGATTGCC -3’
mlrA-CTD	5’- GTAC***GAATTC***AGCTGCGAACGTGGATCAAAGAGCGCGG -3’<br>5’-GCTA***CTCGAG***TTAAATGCCGAGTGGGAAAATATCATGGCC-3’
bluR	5’- CG***GAATTC***AGGCTTATTACAGCATTGG -3’<br>5’- CG***CTCGAG***TTAGGGGGCATGAAAGATG -3’
bluR-NTD	5’- CG***GAATTC***AGGCTTATTACAGCATTGG -3’<br>5’- CG***CTCGAG***TCAGCGGGAGGTGTTGTGATCAAG -3’
bluR-CTD	5’- CG***GAATTC***AGGATACGGAAGATGACTGGAGCCGC -3’<br>5’- CG***CTCGAG***TTAGGGGGCATGAAAGATG -3’
rcsC	5’- CGC***GAATTC***AGGAACTGGCGCATACCTATCTC -3’<br>5’- CGC***CTCGAG***TCACGAATCCCGCGATTTCCTGAC -3’

IX. Primers used for cloning and mutagenizing *bluF* on pQE30Xa-LacIq

bluF-Phos-fw<br>*bluF*-HindIII-rev	5'- Phos-ATGCTTACCACCCTTATTTATC -3'<br>5'- GAC***AAGCTT***TTATTTTTCTCTGGCCACGCTATG -3'
H177Q	5'- TTTGCCTTT**CAG**CCTATTGTC -3'<br>5'- GACAATAGG**CTG**AAAGGCAAA -3'
H306D	5'- GCAATTGAT**GAC**TTTGGCGCA -3'<br>5'- TGCGCCAAA**GTC**ATCAATTGC -3'
M362E, A365E	5'- GTCAGTGCT**GAA**GGCGTG**GAA**ACACCAGAA -3'<br>5'- TTCTGGTGT**TTC**CACGCC**TTC**AGCACTGAC -3'
S328D	5'- ATTAAAATC**GAC**CAGGAATTG -3'<br>5'- CAATTCCTG**GTC**GATTTTAAT -3'
T247N	5'- TTACCTATG**AAC**CTGGTTAAC -3'<br>5'- GTTAACCAG**GTT**CATAGGTAA -3'

X. Primers used for cloning of *mlrA* into pETDuet

mlrA-NdeI	5'- GTC***CATATG***GCGCTTTACACAATTGGTG-3'
mlrA-XhoI	5'- GCT***CTCGAG***AATGCCGAGTGGGAAAATATCATGG-3'

Supplementary References

Barends, T.R., Hartmann, E., Griese, J.J., Beitlich, T., Kirienko, N.V., Ryjenkov, D.A., Reinstein, J., Shoeman, R.L., Gomelsky, M., and Schlichting, I. (2009) Structure and mechanism of a bacterial light-regulated cyclic nucleotide phosphodiesterase. *Nature* 18: 1015-1018.

Brocklehurst, K.R., Hobman, J.L., Lawley, B., Blank, L., Marshall, S.J., Brown, N.L., and Morby, A.P. (1999) ZntR is a Zn(II)-responsive MerR-like transcriptional regulator of *zntA* in *Escherichia coli*. *Mol. Microbiol.* 31: 893-902.

Brown, P.K., Dozois, C.M., Nickerson, C.A., Zuppardo, A., Terlonge, J., and Curtiss III, R. (2001) MlrA, a novel regulator of curli (Agf) and extracellular matrix synthesis by *Escherichia coli* and *Salmonella enterica* serovar typhimurium. *Mol. Microbiol.* 41: 349-363.

Larkin, M.A., Blackshields, G., Brown, N.P., Chenna, R., McGettigan, P.A., McWilliam, H., Valentin, F., Wallace, I.M., Wilm, A., Lopez, R., Thompson, J.D., Gibson, T.J., and Higgins, D.G. (2007) Clustal W and Clustal X version 2.0. *Bioinformatics* 23: 2947-2948.

Letunic, I., Doerks, T., and Bork, P. (2012) SMART 7: recent updates to the protein domain annotation resource. *Nucl. Acids Res.* 40: D302-D305.

Nunoshiba, T., Hidalgo, E., Amábile-Cuevas, C.F., and Demple, B. (1992) Two-stage control of an oxidative stress regulon: the *Escherichia coli* SoxR protein triggers redox-inducible expression of the *soxS* regulatory gene. *J. Bacteriol.* 174: 6054-6060.

Outten, F.W., Outten, C.E., Hale, J., and O'Halloran, T.V. (2000) Transcriptional activation of an *Escherichia coli* copper efflux regulon by the chromosomal MerR homologue, CueR. *J. Biol. Chem.* 275: 31024-31029.

Rao, F., Yang, Y., Qi, Y., and Liang, Z.X. (2008) Catalytic mechanism of c-di-GMP specific phosphodiesterase: a study of the EAL domain-containing RocR from *Pseudomonas aeruginosa*. *J. Bacteriol*. 190: 3622-3631.

Schmidt, A.J., Ryjenkov, D.A., and Gomelsky, M. (2005) The ubiquitous protein domain EAL is a cyclic diguanylate-specific phosphodiesterase: enzymatically active and inactive EAL domains. *J. Bacteriol*. 187: 4774-4781.

Tschowri, N., Busse, S., and Hengge, R. (2009) The BLUF-EAL protein YcgF acts as a direct anti-repressor in a blue light response of *E. coli*. *Genes Dev*. 23: 522-534.
